# Burden of gout among different WHO regions, 1990–2019: estimates from the global burden of disease study

**DOI:** 10.1038/s41598-024-61616-z

**Published:** 2024-07-10

**Authors:** Shoheera Punjwani, Chinmay Jani, Weitao Liu, Loukas Kakoullis, Ingrid Salciccioli, Omar Al Omari, Armaan Merchant, Harpreet Singh, Dominic Marshall, Joseph Shalhoub, Justin D. Salciccioli, Shiv T. Sehra

**Affiliations:** 1 Department of Medicine, Harvard Medical School, Mount Auburn Hospital, 330 Mount Auburn Street, Cambridge, MA 02131 USA; 2 Harvard Medical School, Boston, MA USA; 3 Sylvester Comprehensive Cancer Center at the University’s of Miami, Miami, FL USA; 4 Arlington High School, Arlington, Massachusetts USA; 5 Division of Pulmonary and Critical Care, University of Wisconsin, Milwaukee, WI USA; 6 Critical Care Research Group, Nuffield Department of Clinical Neurosciences, University of Oxford, Oxford, UK; 7 Department of Surgery and Cancer, Academic Section of Vascular Surgery, Imperial College London, London, UK; 8 Imperial Vascular Unit, Imperial College Healthcare NHS Trust, London, UK; 9 Division of Pulmonary and Critical Care, Brigham and Women’s Hospital, Harvard Medical School, Boston, USA; 10 Division of Rheumatology, Harvard Medical School, Mount Auburn Hospital, Cambridge, MA USA

**Keywords:** Gout, Trends, Epidemiology, Incidence, Prevalence, Rheumatology, Gout

## Abstract

The global incidence of gout has increased rapidly, likely secondary to the increase in the prevalence of conditions that predispose to gout, such as obesity. Depending on the population studied, the prevalence of gout ranges from less than 1 to 6.8%. Thus, gout can be a significant burden on healthcare systems. The objective of this study is to observe the trends in the incidence, prevalence, and disability-adjusted life years (DALYs) of gout between 1990 and 2019 globally and in the European Union (EU) 15+ nations. We extracted data from the Global Burden of Disease Study database based on the International Classification of Diseases (ICD) versions 10 and 9. Incidence, prevalence, and disability-adjusted life years (DALYs) were extracted for individual EU15+ countries and globally in males and females between 1990 and 2019. Joinpoint regression analysis was used to describe trends. Between 1990 and 2019, gout prevalence, incidence, and DALYs increased in both males (+ 21.42%, + 16.87%, + 21.49%, respectively) and females (+ 21.06%, + 18.75%, + 20.66%, respectively) globally. The United States of America had the highest increase in prevalence (males: + 90.6%; females + 47.1%), incidence (males: + 63.73%; females: + 39.11%) and DALYs (males: + 90.43%; females: + 42.75%). Incidence, prevalence, and DALYs from gout are increasing worldwide and in most of the EU15+ countries for males and females. Studies have reported the association of gout with comorbidities such as metabolic syndrome, diabetes mellitus, and cardiovascular disease. Health policies and resource allocation are required to increase awareness and modify risk factors globally.

## Introduction

Gout is the most common cause of inflammatory arthritis, affecting approximately 3.9% of the United States (US) population^1^. The global incidence of gout has been increasing rapidly, secondary to the worldwide increase in the prevalence of conditions predisposing to gout’s development^2,3^. Risk factors for the development of gout include obesity, insulin resistance, and metabolic syndrome, hypertension, chronic kidney disease(CKD) among others^3^. Another established risk factor for gout is fructose consumption, and the introduction of high-fructose corn syrup in the manufacturing of soft drinks has coincided with the steepest increase in the prevalence of gout noted in the US. Depending on the population studied, the prevalence of gout varies from less than 1 to 6.8%^1^. A meta-analysis of 71 studies published between 1962 and 2012 regarding the prevalence of gout estimated that the pooled global prevalence of the disease is 0.6% (95% confidence interval 0.4–0.7%); however, a significant degree of heterogeneity was noted among studies^4^. The number of hospitalizations due to gout is also increasing, leading to an increased burden of the disease. It was found that the annual hospitalization rate for gout doubled in the US between 1993 and 2011^5^. Similar increases have been noted in other countries as well, such as Canada, Sweden, and New Zealand^6–8^. On the other hand, gout was also found to have an association with an increase in all-cause and cardiovascular mortality^9^.

Our research group's focus is on understanding the disease burden of gout in EU 15+ countries. Hence, we have utilized this study group, which includes Australia, Austria, Belgium, Canada, Denmark, Finland, France, Germany, Greece, Ireland, Italy, Luxembourg, Netherlands, Norway, Portugal, Spain, Sweden, the United Kingdom (UK), and the US, similar to our previous studies^10–13^. Despite variations in per-capita healthcare expenditure within these nations, research studies have demonstrated that these high-income countries exhibit high similarities in their healthcare system infrastructure and the comprehensiveness of civil registration pertaining to healthcare data, facilitating practical comparisons among them^14^.

We used data obtained from the GBD Study to assess the trends in gout prevalence, incidence, and disability-adjusted life years (DALYs) between the years 1990 and 2019 within the EU15+ countries^10^. Joinpoint regression analysis was used to describe prevalence, incidence, and DALY changes and identify significant trends over the period studied. No such recent analysis comparing trends in this cohort of countries has been performed.

## Methods

### Characteristics of the data source

This observational analysis of gout among EU15+ countries was performed with data collected from the GBD database. The GDB databased is commissioned by the World Health Organization (WHO) and combines 127 countries' datasets and registries that provide epidemiological characteristics (incidence, prevalence, mortality, disability-adjusted life years, years of life lost, etc.) for a variety of health concerns.

Previous publications describe the exact GBD methodology in greater detail^10,11,15^, and we have used this method to describe trends in peripheral arterial disease^13^, abdominal aortic aneurysm^12^, and thyroid cancer^13^. Data sets used by the GBD researchers include variables such as insurance data, admission and outpatient encounter data, and systematic reviews. For gout, the GBD collects data for prevalence and incidence related to the International Classification of Diseases (ICD) codes (code M10 from ICD-10 and code 274 from ICD9). The Bayesian meta-regression is then combined with the data using the DisMod-MR 2.19 tool^16^ which allows for analysis, adjustment for bias, and estimation of diseases with confidence intervals. GBD has different mappings of ICD codes based on incidence or mortality.

### Handling of the GBD data

We extracted age-standardized incidence rates (ASIRs), age-standardized prevalence rates (ASPR), and disability-adjusted life years (DALYs) for gout from the EU15+ countries from 1990 to 2019 using the GDB results tool^17^. Age-standardized rates allow for mathematically adjusting to have the same age structure in each country. This is done by the GDB by calculating a standard population from the United Nations Population Division's World Population Prospects (2012 revision)^18^.

We separated the data for each sex and subsequently calculated both absolute and relative changes for incidence, prevalence, and DALYs from 1990 to 2019 for each country. DALYs include morbidity and mortality data to determine the years of life lost due to premature mortality and lived with a disability. Doing so allows for determining the overall disease burden on a health system^19^. These measures facilitate our understanding of gout's varying temporal impact. Global mean trends are also reported for comparison.

### Statistical analysis

A Joinpoint regression analysis was applied to the data for incidence, prevalence, and DALYs using Joinpoint Command Line Version 4.5.0.1 (provided for free by the United States National Cancer Institute Surveillance Research Program)^20^. The software allows for discerning trends in data over the study period and produces periods where significant trends points are identified. The software also identifies specific points in the overall trends to provide an estimate of the way trends have changed in each country being studied. The simplest model is a straight line as it has no Joinpoints. As more Joinpoints are added, each trend is tested for significance with a Monte Carlo permutation method. The Joinpoint software then computes an estimated annual percentage change (EAPC) with confidence intervals (at 95% confidence) for each line segment. This analysis produces a series of statistically significant Joinpoints for each country with trends represented by an EAPC that may be significant. This allows assessment of trends within time periods to compare countries with each other.

### Ethics approval/consent

Ethics approval was not obtained for this study as this was an observational analysis studying the trends for gout using the GDB database. Written consent was also not necessary as the GDB database is open for data collection and analysis.

## Results

**Figure 1:**
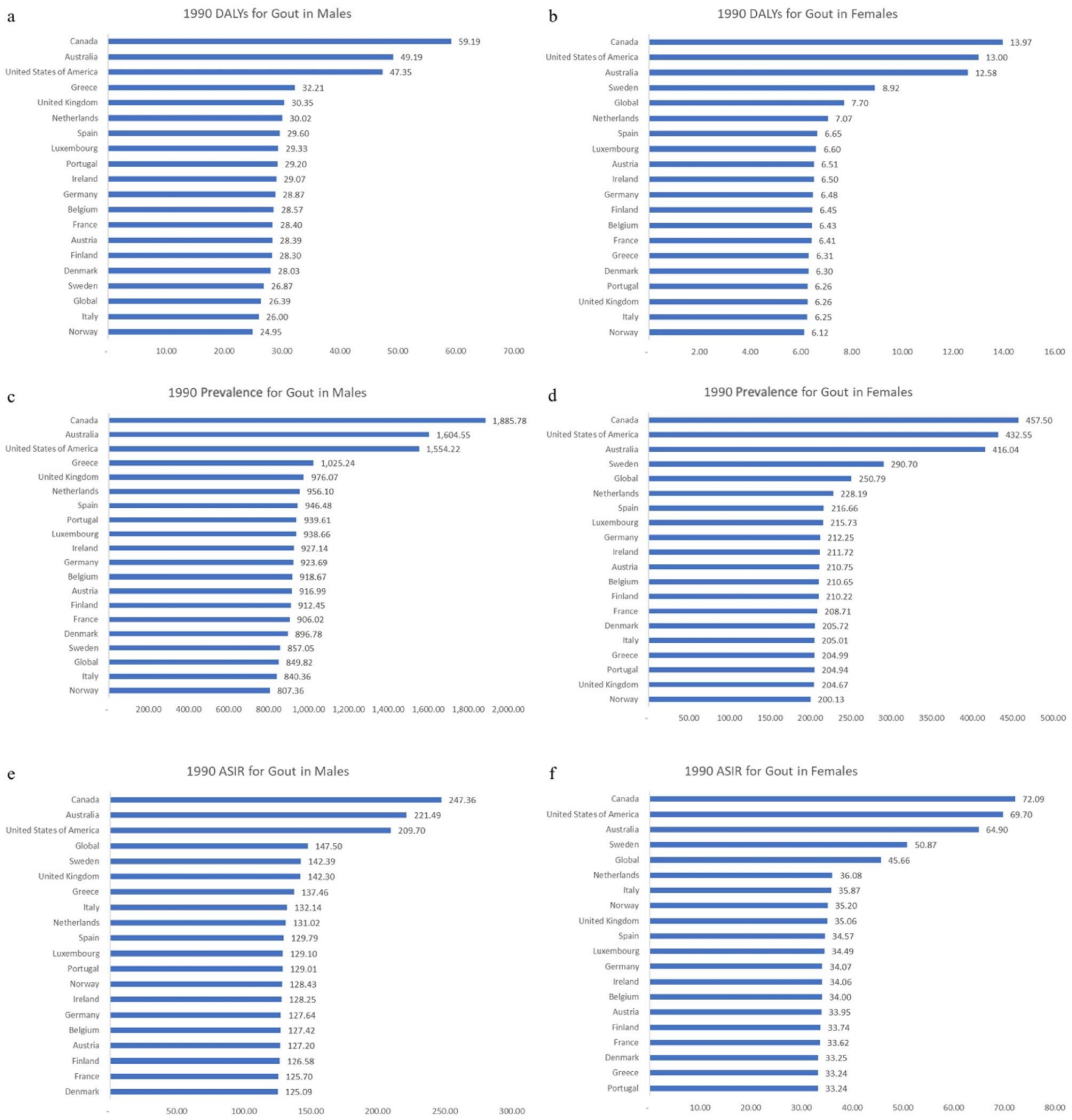
Disability-adjusted life years (DALYs) for males (**a**) and females (**b**), prevalence for males (**c**) and females (**d**), and age-standardized incidence rates (ASIR) for males (**e**) and females (**f**) for gout in the EU15+ countries in 1990. All indices are per 100,000 population.

**Figure 2:**
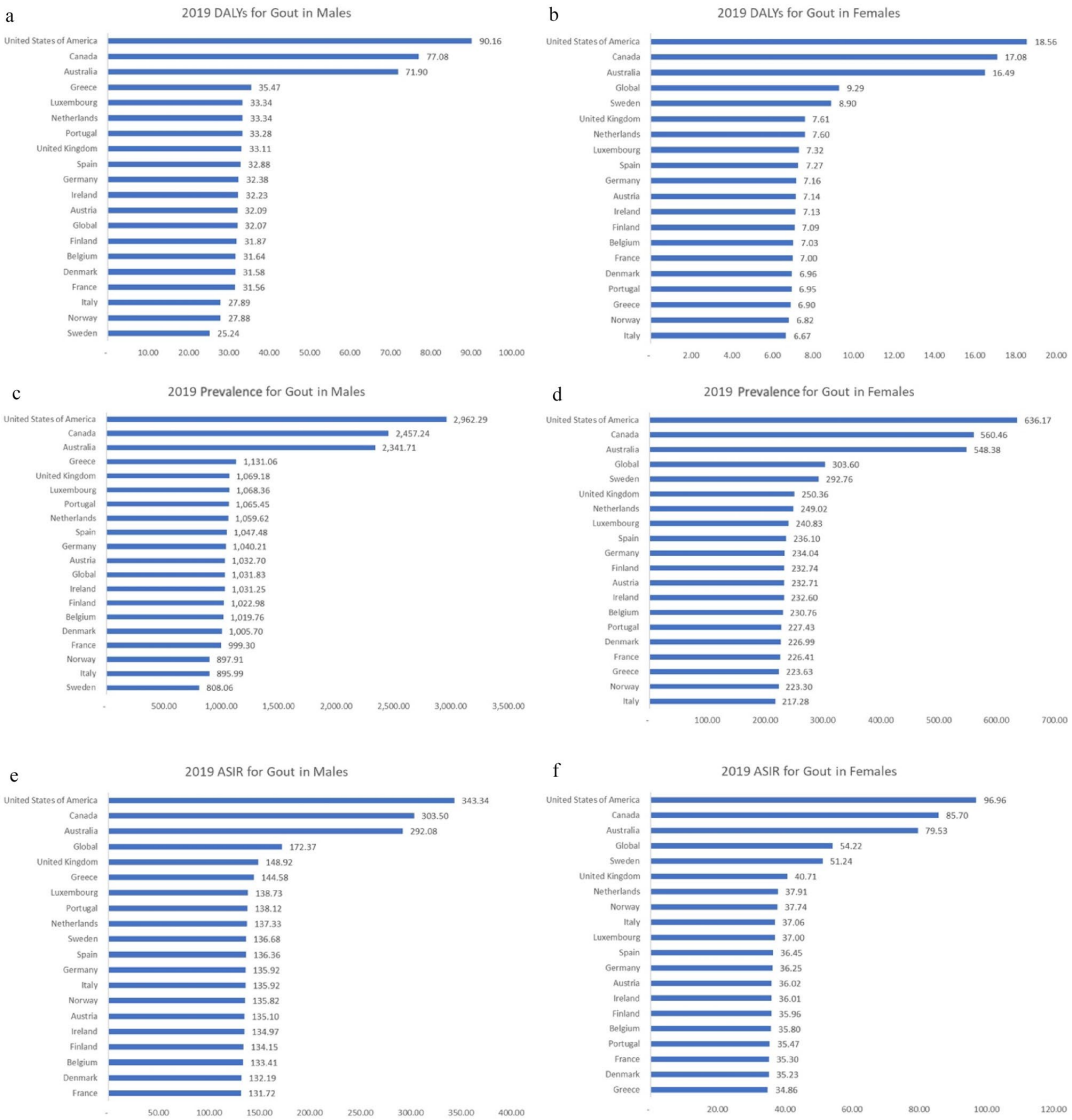
Disability-adjusted life years (DALYs) for males (**a**) and females (**b**), prevalence for males (**c**) and females (**d**), and age-standardized incidence rates (ASIR) for males (**e**) and females (**f**) for gout in the EU15+ countries in 2019. All indices are per 100,000 population.

**Figure 3:**
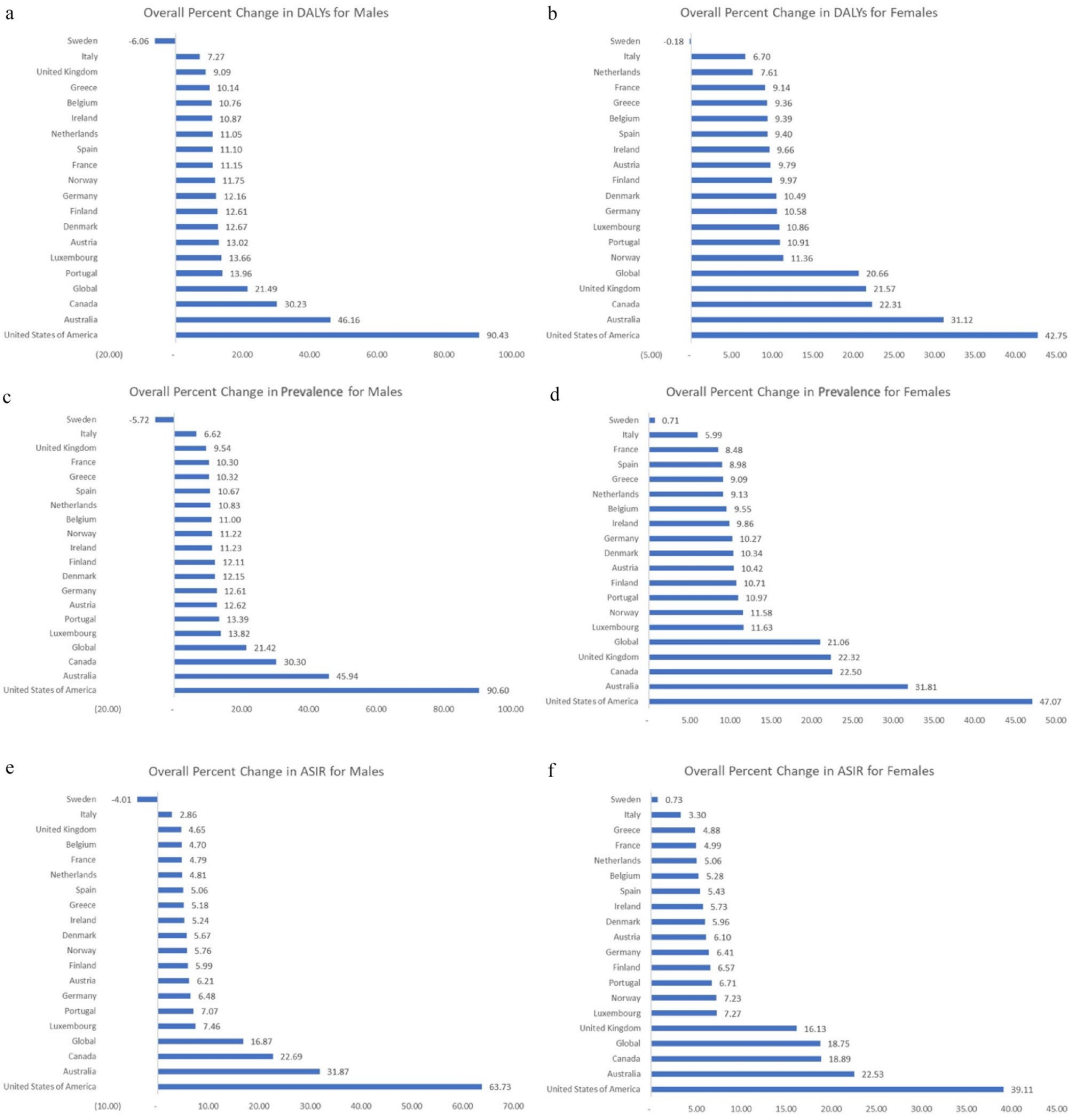
Overall percent change from 1990 to 2019 for disability-adjusted life years (DALYs) for males (**a**) and females (**b**), prevalence for males (**c**) and females (**d**), and age-standardized incidence rates (ASIR) for males (**e**) and females (**f**) for gout in the EU15+ countries.

**Figure 4:**
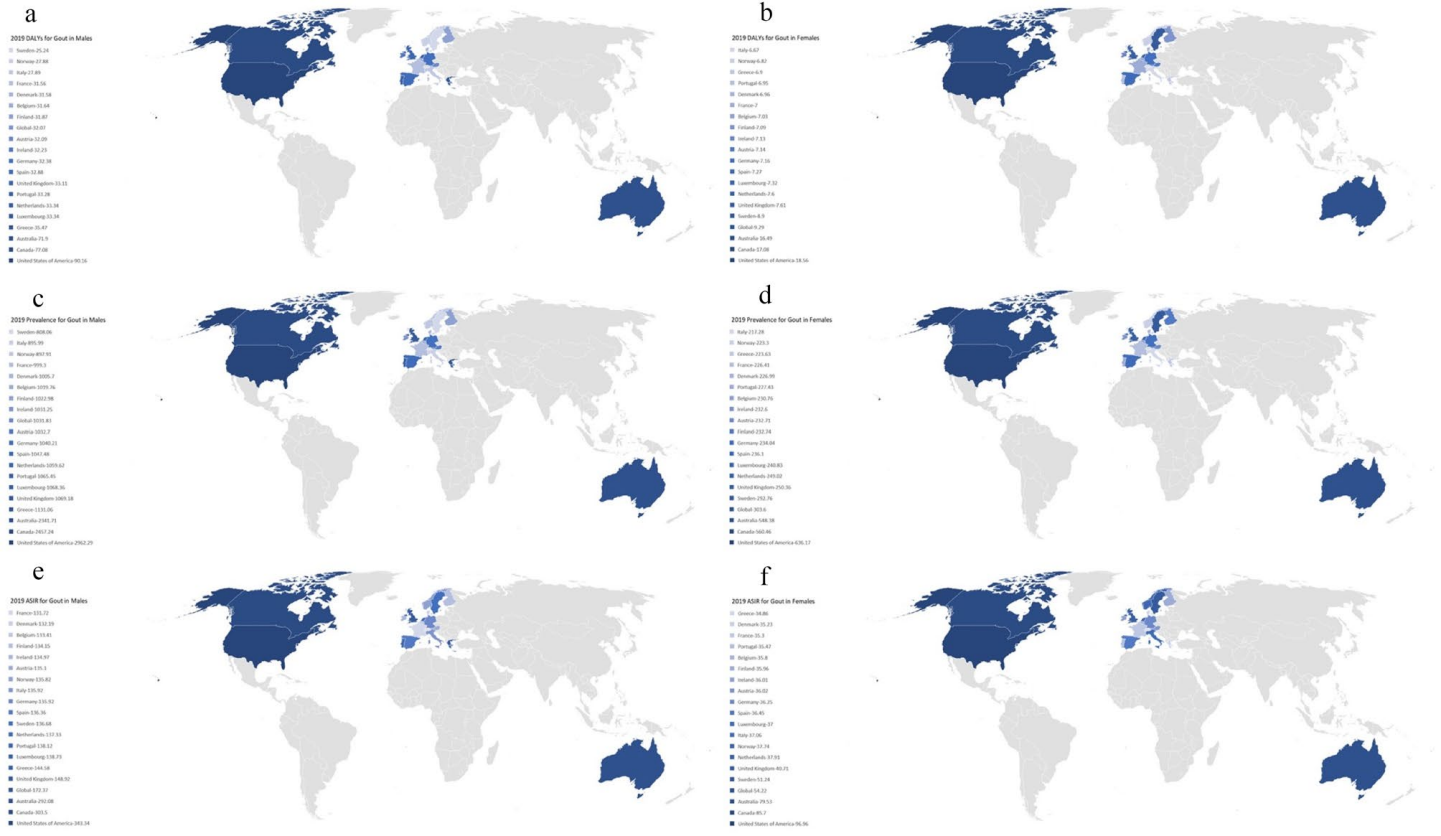
Heat map showing the disability-adjusted life years (DALYs) for males (**a**) and females (**b**), prevalence for males (**c**) and females (**d**), and age-standardized incidence rates (ASIR) for males (**e**) and females (**f**) for gout in the EU15+ countries in 2019. All indices are per 100,000 population.

We observed yearly changes in gout DALYs, prevalence, and ASIR globally and among the EU15+ countries over the 30-year study period from 1990 to 2019. These trends are shown in Tables 1 and 2 and Figs. 1, 2, 3, 4.

**Table 1:** Rates for disability-adjusted life years (DALYs), prevalence, and age-standardized incidence rates (ASIR) in 1990 and 2019 with calculated overall percent changes for gout among males in the European 15+ countries. All indices are per 100,000 population.

Country	DALYs			Prevalence			ASIR		
Male	1990	2019	Percent change	1990	2019	Percent change	1990	2019	Percent change
Global	26.39	32.07	21.49	849.82	1031.83	21.42	147.50	172.37	16.87
Australia	49.19	71.90	46.16	1604.55	2341.71	45.94	221.49	292.08	31.87
Austria	28.39	32.09	13.02	916.99	1032.70	12.62	127.20	135.10	6.21
Belgium	28.57	31.64	10.76	918.67	1019.76	11.00	127.42	133.41	4.70
Denmark	28.03	31.58	12.67	896.78	1005.70	12.15	125.09	132.19	5.67
Finland	28.30	31.87	12.61	912.45	1022.98	12.11	126.58	134.15	5.99
France	28.40	31.56	11.15	906.02	999.30	10.30	125.70	131.72	4.79
Germany	28.87	32.38	12.16	923.69	1040.21	12.61	127.64	135.92	6.48
Greece	32.21	35.47	10.14	1025.24	1131.06	10.32	137.46	144.58	5.18
Ireland	29.07	32.23	10.87	927.14	1031.25	11.23	128.25	134.97	5.24
Italy	26.00	27.89	7.27	840.36	895.99	6.62	132.14	135.92	2.86
Luxembourg	29.33	33.34	13.66	938.66	1068.36	13.82	129.10	138.73	7.46
Netherlands	30.02	33.34	11.05	956.10	1059.62	10.83	131.02	137.33	4.81
Norway	24.95	27.88	11.75	807.36	897.91	11.22	128.43	135.82	5.76
Portugal	29.20	33.28	13.96	939.61	1065.45	13.39	129.01	138.12	7.07
Spain	29.60	32.88	11.10	946.48	1047.48	10.67	129.79	136.36	5.06
Sweden	26.87	25.24	-6.06	857.05	808.06	-5.72	142.39	136.68	-4.01
United Kingdom	30.35	33.11	9.09	976.07	1069.18	9.54	142.30	148.92	4.65
Canada	59.19	77.08	30.23	1885.78	2457.24	30.30	247.36	303.50	22.69
United States of America	47.35	90.16	90.43	1554.22	2962.29	90.60	209.70	343.34	63.73

**Table 2:** Rates for disability-adjusted life years (DALYs), prevalence, and age-standardized incidence rates (ASIR) in 1990 and 2019 with calculated overall percent changes for gout among females in the European 15 + countries. All indices are per 100,000 population.

Country	DALYs			Prevalence			ASIR		
Female	1990	2019	Percent change	1990	2019	Percent change	1990	2019	Percent change
Global	7.70	9.29	20.66	250.79	303.60	21.06	45.66	54.22	18.75
Australia	12.58	16.49	31.12	416.04	548.38	31.81	64.90	79.53	22.53
Austria	6.51	7.14	9.79	210.75	232.71	10.42	33.95	36.02	6.10
Belgium	6.43	7.03	9.39	210.65	230.76	9.55	34.00	35.80	5.28
Denmark	6.30	6.96	10.49	205.72	226.99	10.34	33.25	35.23	5.96
Finland	6.45	7.09	9.97	210.22	232.74	10.71	33.74	35.96	6.57
France	6.41	7.00	9.14	208.71	226.41	8.48	33.62	35.30	4.99
Germany	6.48	7.16	10.58	212.25	234.04	10.27	34.07	36.25	6.41
Greece	6.31	6.90	9.36	204.99	223.63	9.09	33.24	34.86	4.88
Ireland	6.50	7.13	9.66	211.72	232.60	9.86	34.06	36.01	5.73
Italy	6.25	6.67	6.70	205.01	217.28	5.99	35.87	37.06	3.30
Luxembourg	6.60	7.32	10.86	215.73	240.83	11.63	34.49	37.00	7.27
Netherlands	7.07	7.60	7.61	228.19	249.02	9.13	36.08	37.91	5.06
Norway	6.12	6.82	11.36	200.13	223.30	11.58	35.20	37.74	7.23
Portugal	6.26	6.95	10.91	204.94	227.43	10.97	33.24	35.47	6.71
Spain	6.65	7.27	9.40	216.66	236.10	8.98	34.57	36.45	5.43
Sweden	8.92	8.90	-0.18	290.70	292.76	0.71	50.87	51.24	0.73
United Kingdom	6.26	7.61	21.57	204.67	250.36	22.32	35.06	40.71	16.13
Canada	13.97	17.08	22.31	457.50	560.46	22.50	72.09	85.70	18.89
United States of America	13.00	18.56	42.75	432.55	636.17	47.07	69.70	96.96	39.11

### Trends in disability-adjusted life years (DALYs) due to gout

DALYs due to gout were noted to increase in 18/19 countries among males (Fig. 3). The US was noted to have the highest increase in DALYs (+ 90.4%), followed by Australia (+ 46.2%) and Canada (+ 30.2%) among males. Italy (+ 7 0.3%), the UK (+ 9.1%), and Greece (+ 10.1%) were noted to have the smallest increases in DALYs from gout in males. Sweden was noted to have a decrease in DALYs from gout (− 6.1%) in males. DALYs among females due to gout were noted to increase in 18/19 countries. The US was noted to have the highest increase in DALYs (+ 42.7%), followed by Australia (+ 31.1%) and Canada (+ 22.3%) among females. Italy (+ 6.7%), Netherlands (+ 7.6%), and France (+ 9.1%) were noted to have the smallest increases in DALYs from gout among females. Sweden was noted to have a decrease in DALYs from gout (− 0.18%) in females.

### Trends in gout prevalence

Prevalence of gout was noted to increase in 18/19 countries amongst males (Fig. 3). The greatest increase in prevalence was in the US (+ 90.6%), Australia (+ 45.9%), and Canada (+ 30.3%) in males. The smallest increase was in Italy (+ 6.6%), the UK (+ 9.54%), and France (+ 10.3%) in males. Sweden was noted to have a decrease in the prevalence of gout amongst men (− 5.72%). Among females, the prevalence of gout increased in all 19 countries. The US (+ 47.1%), Australia (+ 31.8%), and Canada (+ 22.5%) were noted to have the biggest increases in prevalence among females, whereas Sweden (+ 0.71%) was noted to have the smallest increase in prevalence among females.

### Trends in gout incidence

The incidence of gout increased in 18/19 countries in males. (Fig. 3) The greatest increase in age-standardized incidence rate (ASIR) was in the US (+ 63.7%), Australia (+ 31.9%), and Canada (+ 22.75%) in males while the smallest increase was in Italy (+ 2.9%), UK (+ 4.6%), and Belgium (+ 4.7%) in males. Sweden was noted to have a decrease (− 4.0%) in the ASIR in males. The incidence of gout increased in all 19 countries in females. The greatest increase in ASIR was in the US (+ 39.1%), Australia (+ 22.5%), and Canada (+ 18.9%) in females, while the smallest increase was in Sweden (+ 0.73%).

### Joinpoint regression for DALYs

**Figure 5:**
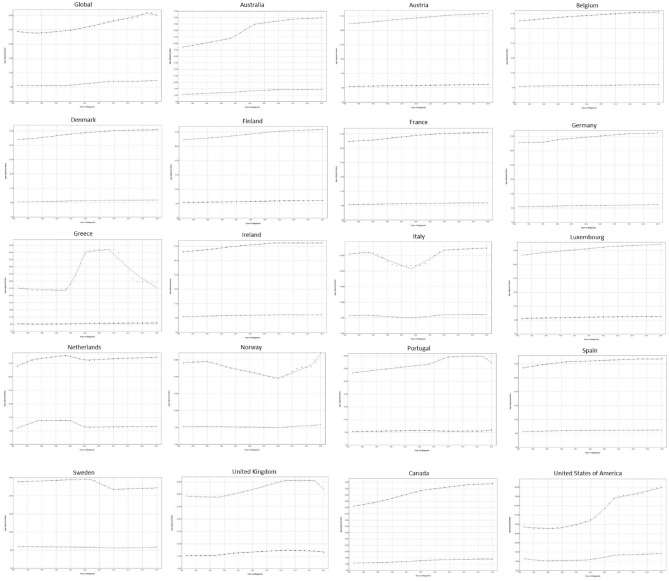
Trends in disability-adjusted life years (DALYs) per 100,000 for gout in the EU15+ countries between 1990 and 2019 as per the Joinpoint regression analysis. Top line indicates males; bottom line indicates females.

Joinpoint regression of DALYs (Fig. 5) showed that globally that the EAPCs for DALYs in men were decreasing in the first period (− 0.6% between 1990 and 1994) and increased in the second and third periods by 0.6% and 1.2%, respectively, only to start decreasing in the final period by − 0.7% annually. Regarding women, DALYs were initially decreasing by − 0.1% and were subsequently either stable (0% between 2009 and 2015) or increasing (2% in the second period and 0.7% in the final period).

During the first period, the greatest increases in DALY EAPCs were noted in the Netherlands for both genders (2.5% in men and 8.1% in women), whereas the greatest declines were found in Greece for men (− 0.5%) and in the USA for women (− 5.1%). In the second period, the greatest increases were seen in Greece for men (17.5%) and the UK for women (3.5%), whereas the greatest declines were noted in Italy, for both men and women (− 2.5 and − 1.5%, respectively). During the third period, the greatest increases for both genders were noted in the USA (7.7% in men and 6.7% in women), and the greatest declines were seen in the Netherlands (− 1.3% in men and − 9% in women). Finally, during the last period, the greatest increases in EAPCs for both men and women were noted in Norway (6.2% and 3.2%, respectively), while the greatest declines were seen in Greece for men (− 6%) and the UK for women (-1%).

### Joinpoint regression for prevalence

**Figure 6:**
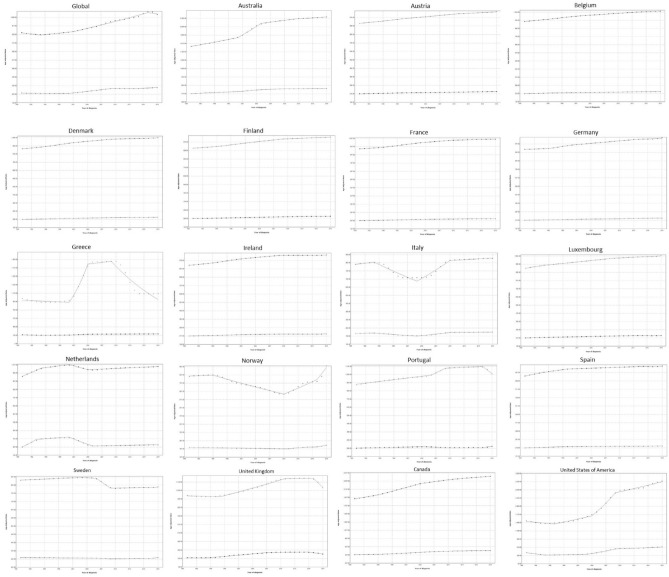
Trends in prevalence per 100,000 for gout in the EU15+ countries between 1990 and 2019 as per the Joinpoint regression analysis. Top line indicates males; bottom line indicates females.

Joinpoint regression analysis demonstrated that the prevalence of gout (Fig. 6) has been somewhat stable for men, with EAPCs ranging between − 0.7 and 1.3%, depending on the different study periods. For women, prevalence had a decade of negative EAPC of − 0.1% between 1990 and 2000, but since then has been mostly steady or increasing, with EAPCs ranging from 0 to 2%.

In the first period, the highest EAPCs for both men and women were noted in the Netherlands (2.1 and 10.1%, respectively), whereas the lowest were noted in Greece for men (− 0.5%) and in the US for women (− 5.2%). During the second study period, the highest EAPC for men was noted in Greece (17.7%) and in Australia in women (2.3%). The lowest EAPCs for both genders were noted in Italy, with prevalence decreasing by − 2.5% in men and − 1.6% in women. The US had the highest EAPCs for both genders during the third period, with prevalence increasing by 8% in men and 7% in women, while the UK and the Netherlands had the lowest in men (− 4.8%) and women (− 6.3%), respectively. During the last study period, the US had the largest EAPC in men (1.4%) while Norway had the highest EAPC in women (3.4%); Greece and the UK had the lowest EAPCs for men (− 6%) and women (− 2.9%), respectively.

### Joinpoint regression for ASIR

**Figure 7:**
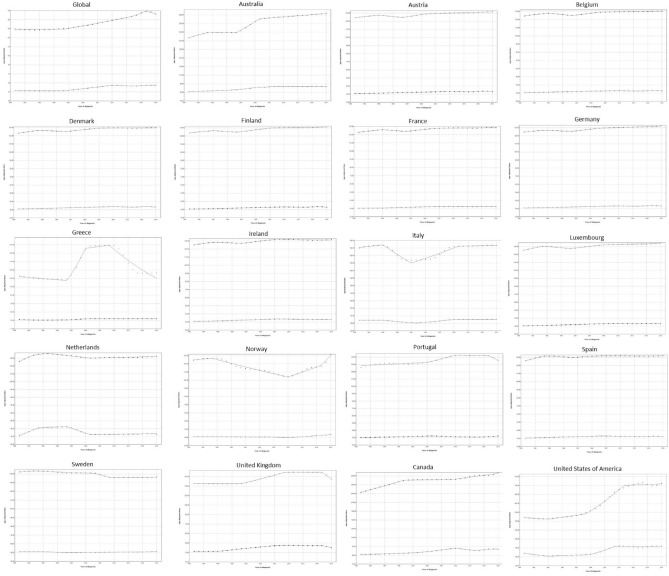
Trends in age-standardized incidence rates (ASIR) per 100,000 for gout in the EU15+ countries between 1990 and 2019 as per the Joinpoint regression analysis. Top line indicates males; bottom line indicates females.

Joinpoint regression analysis demonstrated that globally, ASIR for males did not increase significantly from 1990 to 2000 (EAPC 0.1%; 95% CI 0–0.1; p = 0.185), and subsequently entered an accelerating rate of increase, from 1% (p = 0) between 2000 and 2014, to 1.8% (p = 0.004). (Fig. 7) However, during the last period (2017–2019), the ASIR entered a decline with a rate of − 1.3% (p = 0.027). Regarding females, global ASIR was found to decrease in the first period (1990–2000) by—0.1% (p = 0.045), increased in the second period (2000–2010) by an EAPC of 1.9% (p < 0.001), decreased in the third period (2010–2014) by an EAPC of -0.7% (p = 0.015), and increased in the final period (2014–2019) by an EAPC of 0.6% (p = 0).

During the first period, the highest EAPC for both men (2%) and women (6%) was noted in the Netherlands, whereas the lowest was noted in Greece (− 0.7%) and the USA (− 3.5%) in men and women, respectively. During the second period, the highest EAPC was noted in Greece for men (12.6%) and in Canada and the UK for women (1.9%), whereas the lowest was noted in Italy for both men (− 3.2%) and women (− 1.6%). In the third period, the USA had the highest EAPC for both men (5.2%) and women (8.3%), whereas the lowest was noted in Sweden for males (− 1.1%) and in the Netherlands for females (− 4.7%). Finally, in the fourth period, the highest EAPC for men was noted in Norway (4.9%), and the highest EAPC for women was noted in Portugal (1.1%). The lowest EAPCs in the last period were noted in Greece and the UK for men (-4.6%) and women (− 3.5%), respectively.

## Discussion

The primary aim of this study was to compare and describe data collected by the global burden of disease study among the EU15+ countries for gout regarding prevalence, incidence, and DALYs. Trends were studied using the GBD study data and Joinpoint regression analysis from 1990 to 2019. The importance of this investigation is to understand the changing burden of gout between countries and regions with the aim of establishing a baseline. Our work has been informed by Dehline et al.^1^, who previously reported an increasing prevalence of gout overall and a higher prevalence of gout in men compared with women. This study builds on the previous work as we observed the rate of change during the study period (1990–2019) for incidence, prevalence, and DALYs.

In this observational study, overall, we found the incidence, prevalence, and DALYs from gout are increasing globally and in majority of the EU15+ countries for both males and females. Similar to our current study, in the previous GBD analysis till 2017, the incidence and prevalence of gout increased globally in 2017 compared to 1990, especially in Australasia, high-income North American and Southern Latin American countries. It was observed that burden of gout was generally the highest in developed regions and countries^21^. The overall multifactorial increase in incidence and prevalence may be explained, in part, by the fact that risk factors that are associated with gout have been up trending worldwide, including obesity, CKD, metabolic syndrome, western life style factors, and use of medications that increase uric acid levels (e.g. aspirin and diuretics)^22–26^.The mechanism behind the association between gout and fructose consumption may be direct or indirect, as fructose increases the risk of developing metabolic syndrome^26,27^.

We also noted that of the individual EU15+ countries, the US, Canada, and Australia consistently had the greatest increases in incidence and prevalence for both males and females. Notably, the US also ranked first in terms of incidence and prevalence of gout among all other countries studied. Besides the rising prevalence of factors mentioned above, questions arise on whether there are other factors in the US that accelerated the rising statistics. One possibility is the race disparity, as in a study from 2011 to 2018, Yokose et al. found Asian and Black people had the higher prevalence of gout compared to White and Hispanic people^28^. While the disparity between Black and White people may be partially explained by sociodemographic factors, the disparity between Asian and White people did not appear to associate with sociodemographic factors^28,29^. Further studies need to be done to investigate the rise of gout related statistics worldwide, especially in the US.

Gout is linked with many other comorbidities such as obesity, hypertension, and metabolic syndrome, which are known to elevate mortality risk^30,31^. Moreover, gout alone has been independently linked to a rise in both all-cause and cardiovascular mortality^9^. As a result, when a previously healthy individual is diagnosed with gout, it should prompt a comprehensive evaluation to screen for additional comorbidities. This approach is essential to recognize and manage potential contributing factors, thereby mitigating the increased mortality risk from both gout and its associated conditions. Concurrently, a new gout diagnosis should trigger a review of the patient's medication regimen. This is especially pertinent because some drugs, like thiazide and loop diuretics, which treat certain comorbidities associated with gout, can independently elevate gout risk^32^.

The high incidence and prevalence of gout in countries like the US, Canada, and Australia account for their top rankings in DALYs. Notably, in 2019, the US experienced an astounding surge in DALYs by 90.43% for men and 42.75% for women, placing it rank first for both genders. This is despite the US ranking first in health care expenditure per capita^33^. However, while a third of US healthcare related spending is allocated to hospital care^33^, conditions like gout are predominantly managed on an outpatient basis. Real world studies showed that suboptimal management of gout is common in clinical practice with lower rate of checking serum urate level and low urate-lowering therapy utilization^34,35^. Given this, research on enhancing efficiency and adherence of outpatient management is warranted and potential evaluation and reallocation of US health care spending might be essential for enhanced efficiency.

Interestingly, we observed that the overall percent change in incidence and prevalence, although increasing, was greater in males than females. This suggests that there are factors other than environmental and behavioral that are driving these differences. Estrogen has been hypothesized to be protective against gout as this hormone has a mild uricosuric effect and increases uric acid excretion^36,37^. As such, previous studies have found that the incidence of gout among pre-menopausal women is significantly lower compared to post-menopausal women^36^.

Given the increase in incidence, prevalence, and DALYs for gout globally and individually for most of the EU15+ countries, the management for improving gout flares and decreasing future flares is imperative. Therapeutic guidelines for gout were recently updated^32^. Acute gout flares are still recommended to be treated with non-steroidal anti-inflammatory agents (NSAIDs) or colchicine unless contraindicated^32^. Oral corticosteroids may be used in patients who are unable to be prescribed NSAIDs or colchicine due to intolerance or contraindications^32^. The first-line drug for lowering urate is allopurinol.

## Limitations

The gold standard for diagnosis of gout is arthrocentesis and visualization of monosodium urate crystals. This is not always performed, and diagnosis is often made by symptoms or patient self-report diagnosis, which is prone to misclassification and recall bias. Countries with lower healthcare infrastructure may have a falsely low incidence and prevalence of gout since patients may be less inclined to seek medical attention due to healthcare costs or lack of healthcare facilities or providers.

The GBD Study collaborators acknowledge the limitations of the use of GDB database^38^ which have been discussed previously^11,13^. Specifically, limitations related to the present study include differences in data coding systems across countries as well as the transition from the ICD 9 to ICD 10 system over the period studied. The reliability of the data from EU15+ rests in the fact that the top performing countries regarding the vital statistics and civil registration were Europe, North America, and Australia^10^. Finally, we stress that this is an observational analysis of the trends in the burden of gout across 30 years in EU15+ countries, from which causal inferences should not be concluded. As with all observational analyses, there are likely contributory confounders that are not fully accounted for by using sex-specific, age-standardized incidence and prevalence rates.

## Conclusion

Globally, there has been a notable rise in the incidence, prevalence, and Disability-Adjusted Life Years (DALYs) associated with gout from 1990 to 2019. Notably, these increases are most significant in the US, Canada, and Australia for both genders among EU15+ countries. This surge can be attributed to the rise in comorbid conditions known to be precursors to gout, including obesity, insulin resistance, metabolic syndrome, hypertension, and renal diseases. It is also evident that males bear a disproportionately higher burden of the disease, a phenomenon potentially underlined by the uricosuric effects of estrogen present in females. The gender disparity underscores the need for gender-specific preventative and therapeutic interventions. Considering the unique epidemiological variations from country to country, it's crucial to implement measures tailored to each country's specific data. Enhanced clinical monitoring and strategies to improve ULT adherence are essential steps in reducing gout prevalence and enhancing the future quality of life for patients.

## Data Availability

The datasets generated and analyzed during the current study are available in the GBD Database, which can be found at the following website: https://vizhub.healthdata.org/gbd-results/. ICD 10 code M10, ICD9 code 274. Data was extracted for prevalence, incidence, and disability-adjusted life years for the years 1990 to 2019 and subsequently analyzed.
